# Abscisic Acid-Stress-Ripening Genes Involved in Plant Response to High Salinity and Water Deficit in Durum and Common Wheat

**DOI:** 10.3389/fpls.2022.789701

**Published:** 2022-02-16

**Authors:** Ines Yacoubi, Agata Gadaleta, Nourhen Mathlouthi, Karama Hamdi, Angelica Giancaspro

**Affiliations:** ^1^Laboratoire de Biotechnologie et Amélioration des Plantes, Centre de Biotechnologie de Sfax, Sfax, Tunisia; ^2^Department of Agricultural and Environmental Sciences (DiSAAT), University of Bari Aldo Moro, Bari, Italy

**Keywords:** abscisic acid-stress-ripening, ASR, *TtASR*, *TaASR*, durum wheat, common wheat, high salinity, drought

## Abstract

In the dry and hot Mediterranean regions wheat is greatly susceptible to several abiotic stresses such as extreme temperatures, drought, and salinity, causing plant growth to decrease together with severe yield and quality losses. Thus, the identification of gene sequences involved in plant adaptation to such stresses is crucial for the optimization of molecular tools aimed at genetic selection and development of stress-tolerant varieties. Abscisic acid, stress, ripening-induced (*ASR*) genes act in the protection mechanism against high salinity and water deficit in several plant species. In a previous study, we isolated for the first time the *TtASR1* gene from the 4A chromosome of durum wheat in a salt-tolerant Tunisian landrace and assessed its involvement in plant response to some developmental and environmental signals in several organs. In this work, we focused attention on *ASR* genes located on the homoeologous chromosome group 4 and used for the first time a Real-Time approach to “*in planta”* to evaluate the role of such genes in modulating wheat adaptation to salinity and drought. Gene expression modulation was evaluated under the influence of different variables – kind of stress, ploidy level, susceptibility, plant tissue, time post-stress application, gene chromosome location. *ASR* response to abiotic stresses was found only slightly affected by ploidy level or chromosomal location, as durum and common wheat exhibited a similar gene expression profile in response to salt increase and water deficiency. On the contrary, gene activity was more influenced by other variables such as plant tissue (expression levels were higher in roots than in leaves), kind of stress [NaCl was more affecting than polyethylene glycol (PEG)], and genotype (transcripts accumulated differentially in susceptible or tolerant genotypes). Based on such experimental evidence, we confirmed Abscisic acid, stress, ripening-induced genes involvement in plant response to high salinity and drought and suggested the quantification of gene expression variation after long salt exposure (72 h) as a reliable parameter to discriminate between salt-tolerant and salt-susceptible genotypes in both *Triticum aestivum* and *Triticum durum*.

## Introduction

In the Mediterranean regions characterized by hot and dry climates, wheat is particularly subjected to several abiotic stresses such as heat, drought, and salinity. Salt stress causes osmotic shock at the root level and toxic accumulation in leaves that reduce plant growth, while drought dramatically decreases crop yield and flour quality. Since the last decade, a lot of work has been done to decipher the molecular mechanisms undergoing plant adaptation to such environmental stresses. High salinity and water deficit stimulate a huge change in the expression profiles of different gene classes, most of which are associated with ABA signaling pathways (Rabbani et al., 2003; Shinozaki and Yamaguchi-Shinozaki, 2007). For example, several sequences have been identified as potentially involved in plant drought tolerance in RNA-seq studies carried out in different species (Northey et al., 2016; Lenk et al., 2019), and they belong to metabolic pathways such as brassinosteroid biosynthesis, carbohydrate transport, oxidative stress, and plant cell wall modification (Shabir et al., 2013). However, experimental evidence for the involvement of up- and down-regulated genes in plant stress adaptation remains unclear for most of them, thus representing an essential step to gain the challenging development of new stress-tolerant and more productive varieties. Abscisic acid-stress-ripening (ASR) is a class of plant-specific genes firstly identified by differential screening of a tomato (*Solanum lycopersicum L.*) cDNA library from stressed leaves (Iusem et al., 1993). The ASR protein family consists of small polypeptides isolated in almost all plant species including gymnosperms (*Ginkgo biloba*, Chang et al., 1996), monocots [*Brachypodium distachyon* (Wang et al., 2016); *Oryza sativa L.* (Philippe et al., 2010); maize (Virlouvet et al., 2011); sorghum (Virlouvet et al., 2011); barley (Hamdi et al., 2017; Pérez-Díaz et al., 2019); foxtail millet (Feng et al., 2016; Li et al., 2017)], dicots [*Glycine max* (Li et al., 2013); *Solanum tuberosum L.* (van Berkel et al., 1994)]. Interestingly, they are missing in the model plant *Arabidopsis thaliana* (Carrari et al., 2004). The ASR gene family has been largely reported to be involved in plant response to several developmental and environmental conditions involving ABA signaling, such as environmental abiotic stresses (drought, salinity, metals, heat, cold) (Cakir et al., 2003; Yang et al., 2005; Henry et al., 2011), physiological processes (development, senescence, ripening) (Yang et al., 2005; Maskin et al., 2007; Chen et al., 2011), and biotic stresses (Dai et al., 2011). Most ASR proteins are either located in the cell nucleus functioning as transcription factors (Feng et al., 2016) and/or in the cytosol with chaperone-like activity as we previously showed for durum wheat and barley (Hamdi et al., 2017). Although the exact physiological function and mechanism of action of ASR genes remain unclear, they are a key component in several plant regulatory networks (Philippe et al., 2010; Gonzàlez and Iusem, 2014). This gene family is gaining a lot of importance during the last years as shown to be associated with the modulation of plant adaptation to high salinity and water loss occurring in both physiological and stressed conditions (Leng et al., 2013). ASR proteins have been found accumulated in tissues like leaves and roots suffering water deficit, or in organs undergoing physiological dehydration such as seeds during the late-maturation phase (Maskin et al., 2007), pollen grains in the drying stage (Wang et al., 2013) or fruit berries during ripening (Maskin et al., 2001). Besides modulating plant adaptation to salinity and water lack, ASR proteins also take part in many other secondary cellular pathways such as hexose transport in grape (Cakir et al., 2003), potato (Dominguez et al., 2013), and tobacco (Frankel et al., 2007), aluminum tolerance in rice (Arenhart et al., 2012), cadmium detoxification in maize (Zhang et al., 2019), and oxidative homeostasis in soybean (Li et al., 2013). The involvement of ASR genes in plant drought and salt tolerance was documented in several species such as *Pinus taeda* (Padmanabhan et al., 1997), *Lilium longiflorum* (Huang et al., 2000), *Vitis vinifera* (Cakir et al., 2003), *Solanum lycopersicum L.* (Kalifa et al., 2004), *Brachypodium distachyon* (Wang et al., 2016), *Oryza sativa L*. (Li et al., 2017), *Zea mays L.* (Liang et al., 2019).

For what concerns *Triticum* species, a *TaASR* sequence was firstly isolated in common wheat cv. Chinese Spring by Hu et al. (2013) and enhanced water deficit tolerance in transgenic tobacco plants. For durum wheat, we have previously isolated for the first time a *TtASR1* sequence in a salt-tolerant Tunisian landrace (Hamdi et al., 2020).

Given the increasing availability of crop genomic sequences, the Genome-Wide Analysis allowed the identification of ASR family members in several species and highlighted their gene structure and their evolution history through many phylogenetic studies (Li et al., 2020; Lin et al., 2021). Given the specific conditions needed for the expression of each ASR member and the high similarity percentage between their sequences, it is difficult to carry out expression studies and draw robust conclusions about the role of every single gene and its involvement in abiotic stress tolerance. The present work is in continuity with our previous study on the ASR gene family in wheat, where the expression of a *TtASR1* gene mapping on the 4AL chromosome was shown to be differently regulated by salt stress in the roots of susceptible and tolerant genotypes (Hamdi et al., 2020). Previously, two cereal ASR proteins were produced, experimentally characterized, and shown to be disordered proteins. The addition of glycerol, which mimics dehydration, triggers a gain of structure in both proteins. The structural transition of ASR could explain their versatile function (Hamdi et al., 2017; Yacoubi et al., 2021). Indeed, cereal ASR proteins were shown to function as chaperone-like proteins improve the viability of *E. coli* under heat and cold stress, and increase the *Saccharomyces cerevisiae* tolerance under salt and osmotic stress (Hamdi et al., 2020).

Here, we focused attention on the homoeologous ASR genes located on chromosome group 4 in durum and common wheat and followed their expression pattern by Real-Time qRT PCR under stressed conditions of water deficit and high salinity, in two genotypes showing contrasting phenotype for drought and salt tolerance. By adopting a suitable experimental design, this study was aimed to identify any influence on ASR expression modulation exerted by different variables – treatment (salt vs. drought), ploidy level (durum vs. common wheat), genotype (susceptible vs. tolerant), organ (roots vs. leaves), time-point (6, 24, and 72 h post-stress application), gene chromosome location (4A/4B for durum wheat and 4A/4B/4D for common wheat) - and eventually identify a suitable parameter to discriminate between susceptible and tolerant genotypes.

## Materials and Methods

### Plant Material and Stress Treatments, RNA Isolation, and cDNA Synthesis

Two tetraploids (*Triticum turgidum L. subsp. durum*; 2n = 4x = 28) and two hexaploids (*Triticum aestivum L.*; 2n = 6x = 42) Tunisian wheat genotypes showing different responses to high salinity and water deficit, were used for gene expression studies. The two common wheat cultivars were Ta001*^T^* (salt-drought tolerant) and Ta002*^S^* (salt-drought susceptible), while the two durum varieties were HmiraK1185757*^T^* (salt-drought tolerant) and HmiraK11835*^S^* (salt-drought susceptible). The four original Tunisian genotypes belong to the wheat collection owned by the “Laboratoire de Protection et d’Amélioration des Plantes” at the “Centre de Biotechnologie de Sfax” (CBS), located in Sfax, Tunisia, and were grown in the fields of such Center. All field studies were conducted in accordance with local legislation. These four genotypes were already evaluated for response to drought and salt stress. In previous works, phenotypic and physiologic traits were scored before and after (salt and drought) stress application. Drought tolerance index (DTI) and salt tolerance index (STI) were calculated and used as criteria for the selection of seeds with contrasting tolerance to drought and salt to carry out the ASR expression studies (Kim et al., 2014, 2016).

All wheat seeds were initially surface sterilized by a.5% NaClO wash for 15 min, rinsed three times in sterile water, and germinated on a wet Whatman paper filter placed in Petri dishes after 2-day culture in the dark. Then, 10-day-old seedlings were undergone stress application. For salinity and drought treatments, seedlings were incubated in 200 mM NaCl (Sigma Aldrich, Burlington, MA, United States) or 15% polyethylene glycol (PEG 6000) (Sigma Aldrich, Burlington, MA, United States), respectively. For RNA isolation, leaves and roots of both susceptible and tolerant genotypes were harvested from both control (not treated) and treated plants at 0-, 6-, 24-, and 72-h post-stress application, then immediately frozen in liquid nitrogen. Total RNA was extracted from approximately 200 mg of frozen tissues according to the Trizol method (Invitrogen, Waltham, MA, United States), then reverse-transcribed into double-stranded cDNA by using the MML-reverse transcriptase (Invitrogen, Waltham, MA, United States) as described in Hamdi et al. (2020). cDNA samples were led to a final concentration of 100 ng/μl, to be used in the following amplification reactions.

### Bioinformatics Analyses

Detailed molecular characterization of the *TtASR1* gene isolated by Hamdi et al. (2020) was obtained by blasting its gene and cDNA sequences (GenBank Accession N. KX660742, KX660744) against the durum wheat cv. Svevo complete genome (Maccaferri et al., 2019) available at the INTEROMICS browser: https://www.interomics.eu/durum-wheat-genome. The matching chromosome scaffold coordinates were put in the INTEROMICS d-gbrowse and all the genes localized on chromosome 4 (4A and 4B) were downloaded, along with their alternative splicing forms and the corresponding protein sequences. Homoeologous *ASR* genes on group 4 chromosomes and their corresponding transcripts in common wheat were retrieved by querying the full genome of *T. aestivum* cv. Chinese Spring annotated at the publicly available Plant Ensembl database: http://plants.ensembl.org/index.html.

All molecular comparisons within ASR gene, transcript, and protein sequences were performed by Multiple Sequence Comparison by Log-Expectation (MUSCLE) alignment software available at: http://www.ebi.ac.uk/Tools/msa/muscle/.

### Primer Synthesis, Experimental Design, and Quantitative Reverse Rranscription-Polymerase Chain Reaction Amplification of *TtASR* and *TaASR* Genes

Specific primer pairs were drawn for quantitative reverse transcription-polymerase chain reaction (qRT-PCR) amplification of *ASR* genes on group 4 chromosomes of Tunisian durum and common wheat varieties. For amplification in the tetraploid genotypes HmiraK11835*^S^* and HmiraK11857*^T^*, gene and cDNA sequences of *TtASR1* isolated by Hamdi et al. (2020; GenBank Accession KX660742, KX660744) were N-blasted against cv. Svevo complete genome (Maccaferri et al., 2019) annotated at INTEROMICS and specifically compared with *ASR* sequences on chromosomes 4A and 4B. To thoroughly amplify all the alternative transcript variants, the different splicing forms of Svevo *TtASR-4A* and *TtASR-4B* were multi-aligned and primers opportunely picked in the conserved exon regions. A unique primer pair (P6) could be designed to simultaneously amplify both genes on chromosome group 4 (*TtASR-4A* and *TtASR-4B*).

Primer pairs for qRT-PCR amplification of *ASR* in the Tunisian common wheat varieties Ta001*^T^* and Ta002*^S^* were designed on the *ASR* sequences of *T. aestivum* cv. Chinese Spring annotated at Plant Ensembl database. In particular, two primer pairs were drawn to amplify the homoeologous *TaASR* sequences on 4A (P1) and 4B-4D (P2) chromosomes, respectively. All primers were synthesized by using the Primers3Plus software at: https://www.bioinformatics.nl/cgi-bin/primer3plus/primer3plus.cgi. Oligo quality was checked by using the Primer Structure Analysis tool at: https://www.promix.cribi.unipd.it. The overall experimental design consisted in the expression study of genes *TtASR-4A/4B* in durum wheat, and *TaASR-4A* and *TaASR-4B/4D* in common wheat, in both susceptible and tolerant genotypes, in two organs (leaves and roots), under two abiotic stresses (200 mM NaCl and 15% PEG treatment) at four time-points after stress application (0, 6, 24, and 72 h). In all PCR reactions, *Actin* was used as the internal calibrator to normalize *ASR* expression data. Primers sequence details are reported in Table 1.

**TABLE 1:** Primer features used for abscisic acid-stress-ripening (*ASR*) gene expression study during salt and water stress in wheat.

Wheat genotype	Chr. number	Primer name	Primer sequence (5′-3′)	Annealing temp. (°C)	Amplicon length (bp)
			*F1*:		
			ATGTCGGAGGAGAAGCACCACCA		
*Common*	4A	P1		65	405
			*R1*:		
			TCACTTATGGAACGCACGTAACG		

*Common*	4B		*F2*:		411
			GAGGAGAAGCACCACCACCACC	65	
		P2			
*Common*	4D		*R2*:		408
			CTAGCCGAAGTGGTGGTGCTTCTTC		

*Durum*	4A		*F6*:		411
			ATGGCGGAGGAGAAGCACCACCAC		
		P6			
*Durum*	4B		*R5*:	65	417
			CTAGCCGAAGTGGTGGTGCTTCTTC		

For both *ASR* and *Actin* genes, primer concentrations and annealing temperatures were optimized by running preliminary qRT-PCR reactions with different combinations of Forward and Reverse primers in the final mix (300, 500, and 900 nM), and finally choosing those giving the highest endpoint fluorescence, lowest Cq and best amplification curve. Primers specificity was tested by evaluating the melting curves of PCR products at the end of each amplification reaction.

Following optimization of annealing temperature and primer concentration, qRT-PCR reactions were run using Sybr Green^®^ chemistry in a CFX96 Real-Time System (Bio-Rad, Hercules, CA, United States), according to the following thermal profile: 95°C for 3 min, followed by 40 cycles of 95°C for 10 s and 60°C for 30 s. Each experiment was set in a final reaction volume of 10 μl containing 1 μl of a 1:20 cDNA dilution (approximately 100 ng cDNA), 5 μl of SsoFast Sybr Green^®^ SuperMix 10X (Bio-Rad, Hercules, CA, United States), 2 μl of primer mix (For + Rev), and 2 μl of RNase-free ddH_2_O. Each sample was run in triplicate. For each primer pair, two standards (NT, No Template, and NA, No Amplification) were simultaneously run in the same plate of the unknown samples. For both *ASR* and *Actin* genes, reaction efficiency was calculated by running six-point standard curves of threefold cDNA serial dilutions in the same amplification plate of the samples. Expression data were finally analyzed with the CFX ManagerTM 3.1 software (Bio-Rad, Hercules, CA, United States), using the Normalized Expression mode (ΔΔCq) to normalize the target expression with respect to the internal reference and determine *SD*.

## Results

### Primers Design for Quantitative Reverse Rranscription-Polymerase Chain Reaction Amplification of *ASR* Genes in Durum Wheat

*TtASR1* gene and cDNA sequences isolated in the salt-tolerant Tunisian durum wheat landrace Mahmoudi (Hamdi et al., 2020; GenBank Accession KX660742, KX660744) were N-blasted against Svevo complete genome (Maccaferri et al., 2019) annotated at INTEROMICS. The best ranking matches were *TtASR-4A* (TRITD4Av1G160700) and *TtASR-4B* (TRITD4Bv1G043860), respectively showing an identity percentage of 99 and 97%, and including two exons separated by a short intron.

Alignments of Mahmoudi *TtASR1* gene with Svevo *ASR* sequences on chromosomes 4A and 4B are depicted in Supplementary Figures 1a, 2a: sequence of Mahmoudi almost perfectly matched with *TtASR-4A* of Svevo: the two genes showed a similarity percentage of 99%, encountering only one SNP in the first exon and three SNPs in the second exon, with the last two not influencing protein sequence nor function as falling into the stop codon. The present alignment confirmed gene prediction reported by Hamdi et al. (2020): Mahmoudi *ASR* gene accounts for two exons (225 and 186 bp) separated by one short intron (96 bp) having the same features of Svevo (nucleotide sequence, length, GT-AG splicing junctions); the only difference relies upon the stop codon (TGA in Mahmoudi, TAG in Svevo).

Svevo *TtASR-4A* and *TASR1-4B* genes accounted, respectively, for six and five alternative splicing forms annotated at the INTEROMICS database (Supplementary File 1). Among the transcript variants of the *TtASR1-4A* gene, multi-alignment comparison indicated that the cDNA sequence of the Mahmoudi *ASR* gene had the lowest identity percentage with transcript variant n. 6 (94.6%), but an almost perfect correspondence with the splicing form TRITD4Av1G160700.1 (99% identity) (Supplementary Figure 3).

Comparison between Mahmoudi and Svevo was performed also at protein level (Supplementary Figures 1b, 2b). Predicted protein for Mahmoudi (GenBank Accession ASC55654.1) consisted of 136 amino acids including the conserved abscisic acid-water-deficit stress domain (ABA_WDS) typical of the ASR gene family (PFAM PF02496). As shown in Supplementary Figure 1b, ASR proteins of the two durum wheat varieties differed for only two amino acid substitutions, Alanine/Serine at position n.2 and Leucine/Hystidine at position n.134, which did not fall into the functional ABA_WDS domain.

In conclusion, based on these sequence analyses conducted at both gene and protein levels, we can confidently confirm that the prediction of gene and protein structure of the *TaASR1* gene isolated in the Tunisian landrace Mahmoudi (Hamdi et al., 2020) was accurate and corresponded to the durum wheat cv. Svevo *ASR* gene located on chromosome 4A, specifically to the splicing form TRITD4Av1G160700.1.

**FIGURE 1:**
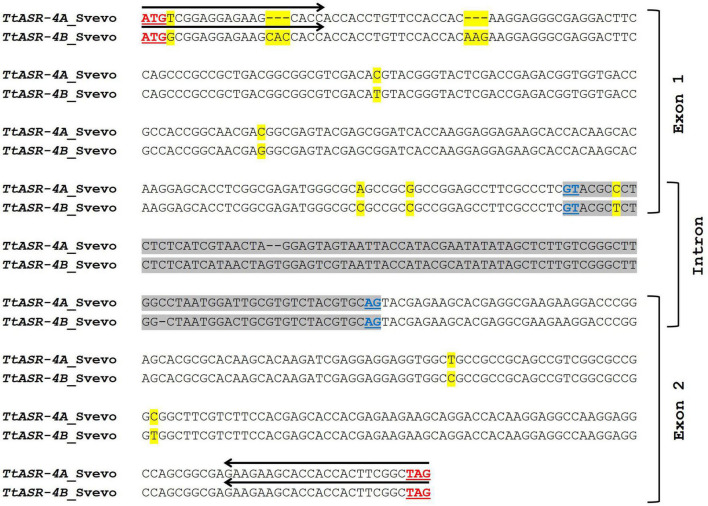
Comparison between *TtASR-4A* (TRITD4Av1G160700) and *TtASR-4B* (TRITD4Bv1G043860) genes of *Triticum durum* cv. Svevo (similarity percentage of 97.3%). The intron sequence is highlighted in gray. Underlined in red are reported the start and stop codons; underlined in dark blue are reported the splice junctions. Black arrows represent Forward and Reverse primers (P6) used for gene amplification in qRT-PCR expression study. SNPs between the two sequences are highlighted in yellow.

Starting from *ASR* sequences on 4A and 4B chromosomes of cv. Svevo, suitable primer pairs were designed to assess the expression of *TtASR* genes in the Tunisian durum wheat varieties Hmira35*^S^* and Hmira57*^R^*. To properly amplify all the alternative transcript variants of each homoeologous gene, the different splicing forms of both *TtASR-4A* and *TtASR-4B* were multi-aligned, and primers opportunely picked in the conserved exon regions. Given the high similarity between homoeologous sequences on 4A and 4B (97.6%, Figure 1), it was not possible to obtain unambiguous genome-specific primers, thus a single primer pair (named P6) was drawn to simultaneously study the expression of both *TtASR-4A* and *TtASR-4B* genes. Oligo sequences were checked for nucleotide composition to avoid the formation of primer-dimers or hairpin loops. Primer sequences and position are reported respectively in Table 1 and Figure 1.

### Primers Design for Quantitative Reverse Rranscription-Polymerase Chain Reaction Amplification of *ASR* Genes in Common Wheat

**FIGURE 2:**
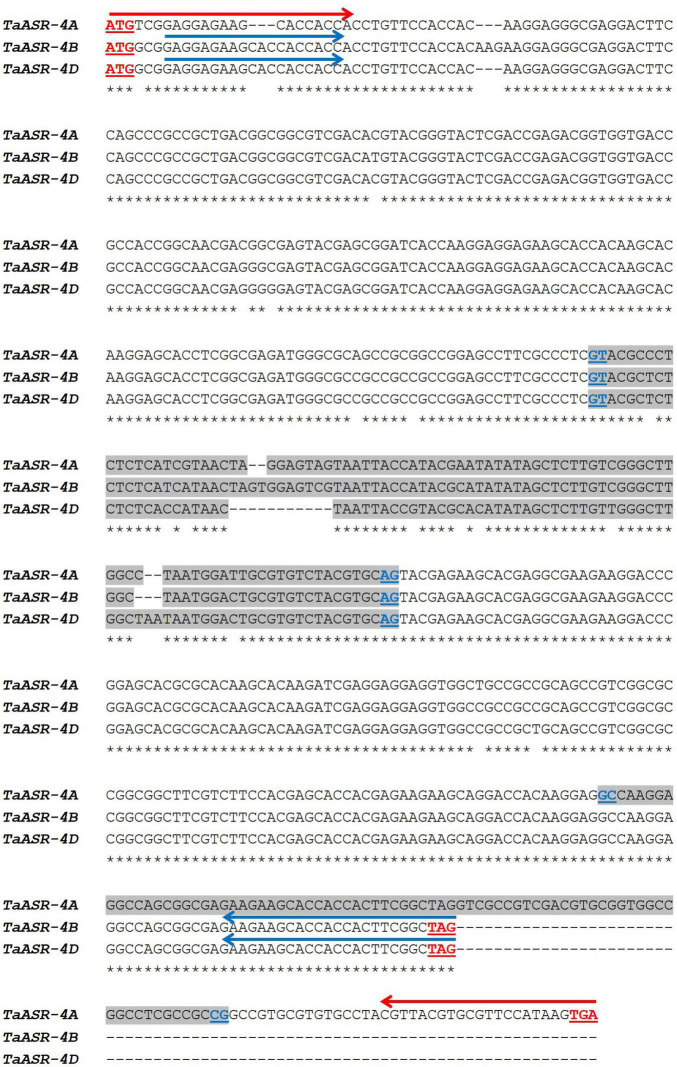
Multiple alignments of common wheat (*Triticum aestivum* cv. Chinese Spring) homoeologous *TaASR* genes on 4A, 4B, and 4D chromosomes. Intron sequences are highlighted in gray. Underlined in red are reported the start and stop codons; underlined in dark blue are reported the splice junctions. Arrows represent primer pairs picked for gene amplification in Real-Time qRT PCR expression studies (red = P1; blue = P2). P1 is the specific primer pair for the A genome; P2 simultaneously amplifies TaASR homoeologous genes from 4B and 4D chromosomes.

Gene and cDNA sequences of *TtASR1* isolated in the salt-tolerant Tunisian durum wheat landrace Mahmoudi (Hamdi et al., 2020; GenBank Accession KX660742, KX660744) were N-blasted against the full genome of *T. aestivum* cv. Chinese Spring annotated at Plant Ensembl database. The homoeologous genes retrieved on group 4 chromosomes - *TaASR-4A* (TraesCS4A02G208400), *TaASR-4B* (TraesCS4B02G112000), and *TaASR-4D* (TraesCS4D02G109500) – were multi-aligned to determine similarity percentage and molecular comparison (Supplementary File 2 and Figure 2). The three genes shared the same GT-AG splicing junctions, but differed for intron sequence, exons number, length, and nucleotide composition (presence of SNPs and in/del mutations). In particular, *TtASR-4B* and *TtASR-4D* showed the same conserved gene structure of the ASR gene family reported in many crops (two exons separated by one intron), whereas *TtASR-4A* included three exons separated by two introns (Figure 2). Similarity percentages among the three homoeologs were the following: 93.8% between *TtASR-4A* and *TtASR-4B*; 93.5% between *TtASR-4A* and *TtASR-4D*; 99.3% between *TtASR-4B* and *TtASR-4D.*

Based on this alignment, primer pairs for amplification of *TaASR* genes on group 4 chromosomes of varieties Ta001*^T^* (salt-drought tolerant) and Ta002*^S^* (salt-drought susceptible) of common wheat, were designed. A specific primer pair (P1) was drawn to specifically amplify *TaASR* on 4A (Figure 2 and Table 1); on the contrary, given the high similarity level between homoeologous sequences on 4B and 4D (99.3%), it was not possible to design genome-specific primers, but a unique primer pair (named P2) was used to simultaneously follow the expression of both genes.

### Setting of Optimal Parameters for qRT Real-Time PCR Amplifications

To obtain specific amplification products of the expected molecular size, primer pairs concentration and annealing temperature for *TtASR*, *TaASR*, and *Actin* genes were optimized by running preliminary qRT-PCR reactions with fluorescent DNA-intercalating SYBRGreen dye (Supplementary Figure 4). By testing different combinations of Forward and Reverse primers in the final mix (300, 500, and 900 nM), a positive correlation between primer concentration and amplification efficiency was observed for all genes at 300 and 500 nM, indicated by a lower Ct value and a higher endpoint fluorescence. However, genes showed a worse amplification profile at 900 nM, giving bad-fitting curves and higher Ct values, probably due to the inhibitory effect exerted by high primer quantity on DNA-polymerase efficiency (Supplementary Figure 4a). Thus, 500 nM was chosen as the optimal concentration for amplifying all genes, being the highest primer quantity ensuring the lowest Ct value and the best amplification curve.

Finally, within the tested range of 55–65°C, we chose 65°C as the finest annealing temperature for all genes, as it allowed us to obtain single amplicons of expected molecular sizes, and optimal melting curves with one single peak representing amplification of the only specific gene product (Supplementary Figure 4b).

### Expression Study of *ASR* Genes in Wheat Under Abiotic Stresses

**FIGURE 3:**
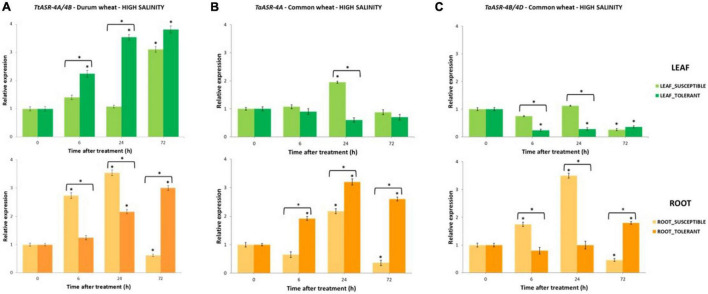
The qRT-PCR time-course expression of *ASR* genes in salt-susceptible and salt-tolerant Tunisian genotypes of tetraploid and hexaploid wheat after 200 mM NaCl application. Gene expression was measured in leaves and roots at 0 (control, not treated, set to 1), 6-, 24-, and 72-h post-treatment. Expression values are reported as fold-changes relative to control, normalized against an internal reference (*Actin*). Data represent the mean ± *SD* of three technical replicates (vertical bars). Asterisks indicate data significantly different between samples and control (* on bars) or between susceptible and tolerant genotypes (* over two bars), according to Student’s *t*-test (*p < 0.05). **(A)** Expression profile of *TtASR* genes from 4A and 4B chromosomes of durum wheat in salt-susceptible (HmiraK11835*^S^*) and salt-tolerant (HmiraK11857*^T^*) genotypes. **(B)** Expression profile of *TaASR* gene from 4A chromosome of common wheat in salt-tolerant (Ta001*^T^*) and salt-susceptible (Ta002*^S^*) genotypes. **(C)** Expression profile of *TaASR* genes from 4B/4D chromosome of common wheat in salt-tolerant (Ta001*^T^*) and salt-susceptible (Ta002*^S^*) genotypes.

**FIGURE 4:**
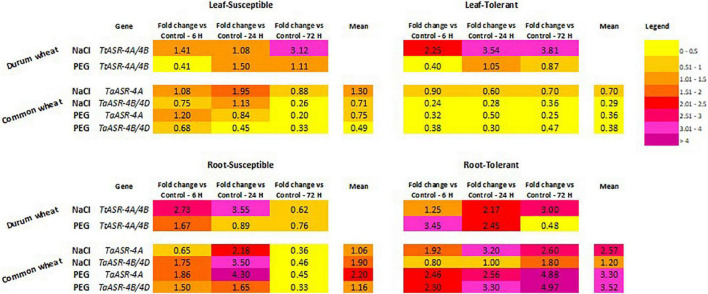
Heat map of *ASR* gene expression levels in leaves and roots of salt-draught susceptible/tolerant Tunisian durum and common wheat genotypes, under abiotic stresses (high salinity: 200 mM NaCl; water deficit: 15% PEG). Colors represent the magnitude of gene expression variation expressed as fold-changes relative to control (0 h, set to 1, not shown) measured at 6-, 24-, and 72-h post-stress application.

**FIGURE 5:**
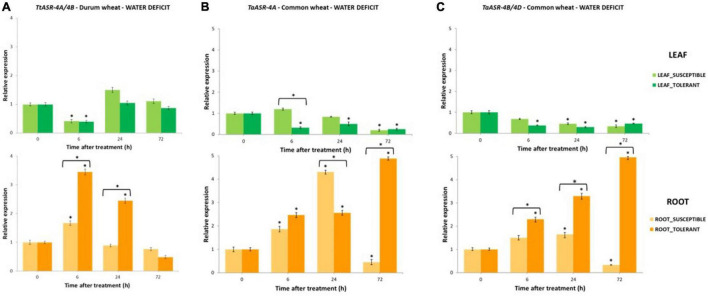
The qRT-PCR time-course expression of *ASR* genes in drought-susceptible and drought-tolerant Tunisian genotypes of tetraploid and hexaploid wheat after 15% PEG application. Gene expression was measured in leaves and roots at 0 (control, not treated, set to 1), 6-, 24-, and 72-h post-treatment. Expression values are reported as fold-changes relative to control, normalized against an internal reference (*Actin*). Data represent the mean ± SD of three technical replicates (vertical bars). Asterisks indicate data significantly different between samples and control (* on bars) or between susceptible and tolerant genotypes (* over two bars), according to Student’s *t*-test (*p < 0.05). **(A)** Expression profile of *TtASR* genes from 4A and 4B chromosomes of durum wheat in drought-susceptible (HmiraK11835*^S^*) and drought-tolerant (HmiraK11857*^T^*) genotypes. **(B)** Expression profile of *TaASR* gene from 4A chromosome of common wheat in drought-tolerant (Ta001*^T^*) and drought-susceptible (Ta002*^S^*) genotypes. **(C)** Expression profile of *TaASR* genes from 4B/4D chromosomes of common wheat in drought-tolerant (Ta001*^T^*) and drought-susceptible (Ta002*^S^*) genotypes.

In the present study, the role of *ASR* genes in plant response to abiotic stresses was evaluated in both tetraploid and common wheat in conditions of high salinity (200 mM NaCl) and water deficit (15% PEG). Responsiveness of *ASR* was preliminarily assessed by measuring transcripts abundance through densitometric analyses of RT-PCR products on leaves and roots of susceptible and tolerant genotypes at 0 (control - not treated), 6-, 24-, and 72-h post-NaCl/PEG application. Gene expression variations during stress treatments were evaluated *in planta* by performing qRT Real-Time PCR on susceptible and tolerant genotypes at the same time-points. Expression values were normalized against an internal reference (*Actin*) and graphically reported as fold-changes relative to controls (Figures 3–5). Statistically significant differences were determined by Student’s *t*-test.

#### Durum Wheat – High Salinity

*ASR* gene expression variation in durum wheat was measured in leaves and roots of Tunisian tetraploid landraces HmiraK11835*^S^* and HmiraK11857*^T^*, respectively salt-susceptible and salt-tolerant. Given the high similarity between *ASR* sequences on 4A and 4B chromosomes (97.3%, Figure 1), it was not possible to design unambiguous genome-specific primers, thus a single primer pair (P6, Figure 1 and Table 1) was used to simultaneously amplify the two homoeologous genes (*TtASR-4A* and *TtASR-4B*). In both plant organs expression levels were measured before stress application and at three time-points after treatment (6, 24, 72 h), then referred to controls and compared between susceptible and tolerant genotypes. Assessment of expression variation after long salt exposure (72 h) is very important because it may be useful to discriminate between salt-tolerant and salt-susceptible genotypes. It could reflect the plant’s long-term adaptation to high salt conditions in contrast to the genes responding to the initial shock of increased salt in the growth medium (Hussein et al., 2014).

Histograms reported in Figure 3A show that *TtASR-4A/4B* genes were constitutively expressed in durum wheat, as some gene activity was detected in both leaves and roots of susceptible and tolerant genotypes also upon non-stressed conditions (0 h, controls, set to 1). However, following NaCl application, *TtASR* genes showed a distinct expression profile in the two organs, in the sense that gene activity seemed not influenced at the leaf level, whereas significant differences in transcript abundance were exhibited between roots of susceptible and tolerant genotypes. In detail, *TtASR-4A/4B* genes were a-specifically induced in leaves, reaching 3.1- and 3.8-fold changes at 72 h after stress application, respectively in the susceptible and tolerant genotype; on the contrary, at the root level, *ASR* was down-regulated in Hmira K11835*^S^* and up-regulated in Hmira K11857*^T^* (with 72 h fold-values of 0.8 and 3, respectively). Such expression pattern is in accordance with what was recently reported in the work by Hamdi et al. (2020), in which an a-specific *ASR* induction was detected in leaves of both susceptible Azizi and tolerant Mahmoudi durum genotypes, whereas a differential regulation was observed in roots (down-regulation in Azizi*^S^* and up-regulation in Mahmoudi*^T^*). Taken together, these observations seemed to suggest that *TtASR* genes are really involved in durum wheat response to high salinity, but their effects are organ dependent as they are exerted only at the root level. In leaves, *ASR* genes seem to be a-specifically induced by salinity, without correlating with salt-tolerance phenotype.

#### Durum Wheat – Water Deficit

Expression profile of *TtASR-4A/4B* genes in response to water deficit in durum wheat was evaluated by using P6 primer pair in the contrasting water-susceptible Hmira K11835*^S^* and water-tolerant Hmira K11857*^T^*, following 15% PEG treatment (Figure 5A). As in salt stress, *ASR* genes were constitutively expressed in absence of any dehydration in both susceptible and tolerant genotypes, but their response to PEG application was different with respect to what was observed upon NaCl treatment. Tolerant genotype showed a gene expression level (average of 6, 24, and 72 h) higher in roots than in leaves, whereas the susceptible line had a comparable gene transcript amount between the two organs. On the contrary, following salt application, the highest gene activity was detected in roots of susceptible genotype and leaves of tolerant one (Figure 4). Differently from high salinity, *ASR* activity in durum wheat seemed not to be influenced by water deficit neither in leaves nor in roots. In leaves, except for a decrease of gene expression at 6 h, *TtASR* activity at 72 h was not significantly different from the control, in both susceptible and tolerant genotypes. Similarly in roots, except for a gene induction immediately after PEG application (6 h), *ASR* response to water deficit was not statistically different in susceptible and tolerant varieties at 72 h, when gene activity in the two genotypes was 0.8 and 0.5% of controls, respectively (Figure 5A).

As already reported for high salinity, our description of *ASR* gene behavior in water deficit conditions is in accordance with what was previously reported by Hamdi et al. (2020), who found an a-specific induction of *TtASR* genes in leaves, and an a-specific down-regulation of the same genes in roots of both drought-susceptible Azizi and drought-tolerant Mahmoudi durum wheat genotypes. *ASR* genes response to high salinity and water deficit in leaves and roots of durum wheat is summarized in Table 2.

**TABLE 2:** Summary of *ASR* genes response to high salinity and water deficit in leaves and roots of Tunisian durum and common wheat varieties.

		Durum wheat		Common wheat		
		Genotype	*TtASR-4A/4B*	Genotype	*TaASR-4A*	*TaASR-4B/4D*
Salt stress (200 mM NaCl)	Leaf	HmiraK11835*^S^*	I	Ta002*^S^*	–	R
		HmiraK11857*^T^*	I	Ta001*^T^*	–	R
	Root	HmiraK11835*^S^*	R	Ta002*^S^*	R	R
		HmiraK11857*^T^*	I	Ta001*^T^*	I	I
Water stress (15% PEG)	Leaf	HmiraK11835*^S^*	I	Ta002*^S^*	R	R
		HmiraK11857*^T^*	-	Ta001*^T^*	R	R
	Root	HmiraK11835*^S^*	R	Ta002*^S^*	R	R
		HmiraK11857*^T^*	R	Ta001*^T^*	I	I

*Gene up- or down-regulation was determined by measuring expression fold-change variation with respect to control in salt-draught susceptible and tolerant varieties, at 72 h after stress application.*

*I, gene induction; R, gene repression; –, not significant variation with respect to control.*

#### Common Wheat-High Salinity

*ASR* gene expression study in common wheat was performed on the two Tunisian cultivars Ta001*^T^* and Ta002*^S^*, salt-tolerant and salt-susceptible, respectively. Two primer pairs were used to specifically amplify the homoeologous *TaASR* genes on 4A (P1) and 4B/4D chromosomes (P2) (Figure 2 and Table 1). Gene transcript levels were measured in leaves and roots of the two hexaploid genotypes before treatment (0 h, control, set to 1) and at 6-, 24-, and 72-h post-stress application (200 mM NaCl). Expression values were normalized against an internal reference (*Actin*) and reported as fold-changes variation relative to controls.

Abscisic *ASR* gene response to high salinity in common wheat is depicted in Figures 3B,C. As in durum wheat, homoeologous *TaASR* genes were constitutively expressed in both organs of hexaploid wheat in absence of any stress application (control relative expression set to 1), and this is because this gene family is involved in several physiological pathways other than a stress response. As for durum wheat, *ASR* transcript levels in roots were higher than in leaves, with average expression values > 1 and ≤1, respectively. Differences between the two organs were observed in both genotypes, but they were more evident in the salt-tolerant variety where gene expression levels were 3.7- and 4.1-fold in roots than in leaves, respectively for *TaASR-4A* and *TaASR-4B*/*4D*. The only exception was represented by the *ASR* gene on the 4A chromosome of salt-susceptible genotype, whose transcript abundance was almost comparable between leaves and roots (Figure 4).

*ASR* response to high salinity in common wheat was similar to durum, in the sense that a significantly different gene expression profile between susceptible and tolerant genotype was detected only at the root level. On the contrary, gene activity seemed not to be influenced by salt concentration in leaves, as indicated by the a-specific response exhibited by both Ta001*^T^* and Ta002*^S^* upon 200 mM NaCl application. Expression profile of *TaASR-4A* gene in leaves is reported in Figure 3B: except for a difference at 24 h (higher transcript abundance in the susceptible variety), final transcript levels of both S and T genotypes were comparable to controls at 72 h. Similar behavior was observed for *TaASR-4B/4D* homoeologous (Figure 3C): following a decrease of transcript levels in the only tolerant variety at 6 and 24 h, gene expression dropped down at 72 h in both genotypes, reaching much lower values than the controls (26 and 36%, respectively in S and T).

In roots, differences in *ASR* expression profile between Ta001*^T^* and Ta002*^S^* upon NaCl application were significant: transcript levels of all the three homoeologous genes were significantly different between S and T genotypes at both 6, 24, and 72 h (Figures 3B,C). In particular, at 72 h, a significant up-regulation was detected in the tolerant line, whereas a down-regulation was observed in the susceptible one, showing final fold-changes of 2.6 vs. 0.4 for *TaASR-4A*, and 1.8 vs. 0.5 for *TaASR-4B/4D*, respectively in Ta001*^T^* and Ta002*^S^*. Such significant accumulation of *ASR* transcripts in the tolerant genotype at 72 h after NaCl application, was clearly discriminating between tolerant and susceptible genotypes.

#### Common Wheat – Water Deficit

The role of *TaASR* genes in response to water deficit in common wheat was investigated by applying 15% PEG to leaves and roots of drought-tolerant Ta001*^T^* and drought-susceptible Ta002*^S^* Tunisian varieties, then following gene expression variation at 6-, 24-, and 72-h post-treatment (Figures 5B,C). By using opportunely designed primer pairs, gene profile was drawn separately for homoeologous sequences on 4A and 4B/4D chromosomes (Figure 2 and Table 1). As expected, we found all the three homoeologs constitutively expressed in non-stressed conditions because *ASR* plays a role in several physiological and developmental cellular processes involving water loss, other than drought. As in durum wheat, the measured activity of the *ASR* gene was higher in roots than in leaves, as indicated by the average relative expression values constantly lower than control in leaves (<1) and higher than control in roots (>1). In particular, the highest difference between the two organs was detected in the drought-tolerant genotype whose transcript levels in roots were about nine times higher than in leaves, for both *TaASR-4A* and *TaASR-4B/4D* homoeologs (Figure 4).

As for high salinity, significant differences in *TaASR* gene expression between susceptible and tolerant genotypes were detected only at the root level, whereas gene activity seemed not to be specifically affected by water deficit in leaves. In fact, in this tissue gene response to PEG treatment was not distinguishable between Ta001*^T^* and Ta002*^S^*, as in both genotypes *ASR* was down-regulated reaching final values (at 72 h) of 0.2 vs. 0.3 of controls for *TaASR-4A* (Figure 5B) and 0.3 vs. 0.5 of controls for *TaASR-4B/4D* (Figure 5C) in susceptible and tolerant lines, respectively. On the contrary, *TaASR* response to PEG was significantly different in roots of Ta001T and Ta002S: all the three homoeologous genes were up-regulated during water deficit in the tolerant genotype, reaching a final relative expression at 72 h of approximately 5 for both *TaASR-4A* (Figure 5B) and *TaASR-4B/4D* (Figure 5C); on the contrary, the three genes were deeply repressed in the susceptible variety showing relative expression values at 72 h of 0.5 for *TaASR-4A* and 0.3 for *TaASR-4B/4D* (Figure 4). ASR genes response to high salinity and water deficit in leaves and roots of common wheat is summarized in Table 2.

## Discussion

Abscisic *ASR* is a small family of hydrophilic proteins isolated in almost all plant species but missing in the model plant *Arabidopsis* (Carrari et al., 2004). Interestingly, no *ASR* orthologs have been identified in organisms outside the plant kingdom. In addition to taking part to plant response to several physiological and environmental processes involving ABA signaling (biotic and abiotic stresses, development, senescence, ripening), ASR proteins accumulate in tissues like leaves and roots during water deficit occurring in both physiological and stressed conditions (Maskin et al., 2001, Maskin et al., 2007; Wang et al., 2013). Identification and experimental validation of candidate genes involved in plant stress adaptation is an essential step to develop novel varieties with enhanced stress tolerance, or design specific molecular tools suitable for marker-assisted selection. In a recent study by Hamdi et al. (2020) a *TtASR1* gene from the 4AL chromosome of durum wheat was isolated for the first time in the salt-tolerant Tunisian landrace Mahmoudi, and its role in response to ABA, PEG, and salt stress was experimentally assessed by qRT-PCR in several plant organs. This gene was also found to improve *E. coli* viability under heat and cold stress and increase *S. cerevisiae*’s tolerance to salt and osmotic stress. A *TaASR* gene was also isolated in the common wheat cv Chinese Spring by Hu et al. (2013) which proved to enhance water deficit tolerance in transgenic tobacco plants. In this work, we focused attention on the homoeologous *ASR* genes mapping on group 4 chromosomes of durum and common wheat, and experimentally investigated their involvement in plant response to increased salinity and drought. An *in silico* bioinformatic analysis identified two ASR members (*TtASR-4A*, TRITD4Av1G160700 and *TtASR-4B*, TRITD4Bv1G043860) in durum wheat, and three ASR homoeologs (*TaASR-4A*, TraesCS4A02G208400; *TaASR-4B*, TraesCS4B02G112000 and *TaASR-4D*, TraesCS4D02G109500) in common wheat. Four of the five identified sequences shared the same gene structure, represented by two exons separated by one intron; only *TaASR-4A* comprised three exons separated by two introns. This very simple molecular structure of *ASR* genes (2 exons – 1 intron) is also shared by other plant species such as tomato (Rossi and Iusem, 1995), rice (Philippe et al., 2010), the genus Musa (plantain and banana, Henry et al., 2011) and the halophyte plant *S. brachiata* (Jha et al., 2009). Previous studies indicated that plant stress-responsive genes completely lacking or with only a few introns could be functional to reduce the time required from transcription to translation, thus ensuring plants with better and faster adaptation abilities in response to changing environmental and habitat conditions (Laha et al., 2020).

In the present study, the involvement of *ASR* gene in plant response to high salinity and water deficit was evaluated *in planta* by following gene expression variation after NaCl/PEG application in leaves and roots of susceptible and tolerant genotypes of durum and common wheat at three-time points after stress application (6, 24, and 72 h). The overall experimental design consisted in studying the expression pattern of 5 ASR genes (*TtASR-4A/4B* in durum wheat and *TaASR-4A*, *TaASR-4B/4D* in common wheat), in four wheat genotypes (susceptible and tolerant varieties of durum and common), in two organs (leaves and roots), under two abiotic stresses (200 mM NaCl and 15% PEG treatment). In this way, we could simultaneously assess the impact of many parameters as any influence on *ASR* activity-dependent on different variables – kind of abiotic stress (salt vs. drought), ploidy level (tetraploid vs. hexaploidy wheat), genotype (susceptible vs. tolerant), and plant organ (leaves vs. roots).

As previously reported in the literature, wheat *ASR* homoeologs located on chromosome group 4 were found constitutively expressed in plant roots and leaves in both stressed and not-stressed conditions. Our results confirmed that such genes are implicated in a series of physiological and developmental cellular processes other than abiotic stresses. The ASR gene family was already shown to be involved in cell elongation and differentiation, pollen maturation, and ripening (Cakir et al., 2003; Yang et al., 2005; Maskin et al., 2007; Chen et al., 2011; Henry et al., 2011). Increasing evidence also assessed ASR proteins’ involvement in plant growth, fruit ripening (Srivastava and Handa, 2005), and tuber development in potatoes (Frankel et al., 2007). Moreover, maize *Asr* genes also seemed to be involved in regulating the biosynthesis of branched-chain amino acids such as Val, Leu, and Ile (Virlouvet et al., 2011).

Expression of *ASR* genes exhibited significant dissimilarities between genotypes (tolerant/susceptible) and depended on the applied stress (salt/drought). By investigating gene activity in both *T. durum* and *T. aestivum*, we found that the expression of the *ASR* gene during abiotic stresses was only partially affected by ploidy level. Moreover, it seemed not to be affected by chromosomal location, too; and this is deducible by the similar behavior of the *ASR* homoeologous gene copies on 4A and 4B/4D chromosomes of common wheat under both salt and drought stress (Figures 3B,C, 4, 5B,C). Worthy of note is that *ASR* gene activity seemed to be more influenced by other variables such as plant tissue (transcripts abundance was higher in roots than in leaves, in both durum and common wheat), treatment (NaCl was more impacting than PEG), and genotype (genes were differentially regulated in susceptible or tolerant varieties).

In both tetraploid and hexaploid wheat marked differences in *ASR* expression were observed between the two analyzed plant organs, in the sense that roots were almost always more responsive than leaves; in the future, this could be very helpful to better decipher the mechanism of action of *ASR* gene in plant cell and target improvement of stress tolerance. In common wheat, strong dissimilarities between roots and leaves were observed for all the three homoeologous genes in both salt and water stress conditions, in both susceptible and tolerant genotypes. Conversely, in durum wheat, a higher gene transcript abundance in roots was detected only in the tolerant genotype under water deficit, and in the susceptible line during salt stress. The higher *ASR* activity in roots with respect to leaves was previously reported in common wheat by Hu et al. (2013), and we could speculate that this is because roots are the first plant organ to suffer and defend against the osmotic stress caused by high salinity and water deficiency. In accordance with the obtained results, rice ASR genes exhibited non-overlapping expression patterns in different tissues and showed different responses to ABA and water stress (Perez-Diaz et al., 2014).

By determining the expression pattern of ASR homoeologs in tetraploid and hexaploid wheat, the present study allowed to investigate any influence of genome ploidy level on *ASR* activity and modulation of plant susceptibility/tolerance to high salinity and drought. Durum and common wheat showed different behavior depending on the applied abiotic stress: the two species shared a similar gene regulation pattern in response to high salinity (gene induction in tolerant genotypes, gene repression in susceptible); on the contrary, they exhibited a different gene profile under drought (similar response to high salinity was observed in the hexaploid genotypes, whereas no responsiveness could be detected in tetraploid ones).

In durum wheat, *TtASR-4A/4B* homoeologs were up-regulated following NaCl application in both leaves and roots, thus we could consider the relative expression value estimated at 72 h post salt treatment in roots as a discriminating variable between salt-tolerant and salt-susceptible genotypes (3 vs. 0.6). On the contrary, expression variation of *TtASR-4A/4B* genes could not be used to discriminate between drought-susceptible and drought-tolerant varieties, as they were repressed in roots of both resistant and sensible genotypes at 72 h post-PEG application. In common wheat, ASR gene expression levels in roots at 72 h post-stress application could be used to discriminate between susceptible and tolerant genotypes in both salt (0.4 vs. 2.6 for *TtASR-4A*) and drought (0.3 vs. 0.5 for *TtASR-4B/4D*) stressed conditions.

Based on such results, we could suggest *ASR* as a robust candidate gene for abiotic stress resistance, and a potential tool for the development of functional markers addressed to salt-tolerant selection in durum and common wheat breeding programs. Similar responsiveness of *ASR* upon stress application was assessed for *CrASR3*, a *Canavalia rosea ASR* gene induced by mannitol in roots (Lin et al., 2021). In rice, some of the putative targets of *ASR5* in roots were reported to be related to Al tolerance, like the ABC transporter. Arenhart et al. (2013) reported a differential pattern in rice where the *OsASR5* gene was induced in roots of Aluminum-tolerant cv. Nipponbare, and not responded in roots of the Al-sensitive cv. Taim. Up-regulation of *ASR* transcripts was also reported in common wheat plants subjected to PEG treatment (Hu et al., 2013). A very recent report demonstrated that overexpressed LEA genes in different organisms resulted in improved tolerance to high salinity and dehydration (Lin et al., 2021).

The experimental evidence collected in the present study based on Real-Time PCR data, strongly suggests that *ASR* genes may be key factors influencing wheat adaptability to high salinity and drought. Indeed, microarray analyses carried out on *Triticum* varieties exhibiting contrasting phenotypes for salt tolerance, indicate that two distinct gene categories take part to plant response to abiotic stresses, acting at different moments: genes expressed under longer stress exposure were hypothesized to reflect a long-term acclimation and plant adaptation to high salt concentration, in contrast to early genes acting only during the initial shock of increased salt concentration in the growth environment (Hussein et al., 2014).

Finally, we interestingly found *ASR* genes more expressed in hexaploid than in tetraploid wheat, in both stressed and not-stress conditions, and we may speculate that this could be due to the presence of an additional homoeologous gene copy located on chromosome 4D of common wheat. These ploidy-dependent *ASR* expression levels, not associated with any stress, may reflect a constitutive difference between hexaploid and tetraploid genotypes which could partially explain the higher salt tolerance of common wheat with respect to durum, reported by Munns and Tester (2008). The impact of polyploidy on plant stress-tolerant phenotype is fully argued in earlier studies on various crops.

Very little work has been reported so far on the experimental validation of *ASR* role in plant adaptation to abiotic stresses in wheat. Based on the empirical evidence collected in this study and on the consistency with the few similar works reported in the literature, we could confidently suggest the real involvement of *ASR* in wheat adaptation to high salinity and water deficit in both *T. aestivum* and *T. durum*, and candidate this gene as a suitable functional molecular tool to target marker-assisted selection (MAS) or develop more tolerant and productive varieties in wheat breeding programs.

## Data Availability Statement

The original contributions presented in the study are included in the article/Supplementary Material, further inquiries can be directed to the corresponding author/s.

## Author Contributions

IY, AGa, and AGi designed the experiments. IY, NM, and KH grew wheat plants on fields, carried out stress treatments, samples collection after stress application, oligonucleotide primer design, RNA isolation, and cDNA synthesis. AGi performed the bioinformatics and molecular analyses, qRT-PCR reactions, and expression data interpretation. AGi and IY wrote the manuscript. AGa, AGi, and IY contributed to data interpretation and designed the experiments. All the authors read and approved the final manuscript.

## Conflict of Interest

The authors declare that the research was conducted in the absence of any commercial or financial relationships that could be construed as a potential conflict of interest.

## Publisher’s Note

All claims expressed in this article are solely those of the authors and do not necessarily represent those of their affiliated organizations, or those of the publisher, the editors and the reviewers. Any product that may be evaluated in this article, or claim that may be made by its manufacturer, is not guaranteed or endorsed by the publisher.
