# Supplementary material for: Abscisic Acid-Stress-Ripening Genes Involved in Plant Response to High Salinity and Water Deficit in Durum and Common Wheat
Source: Front Plant Sci. 2022 Feb 16;13:789701. doi: 10.3389/fpls.2022.789701 (PMC8905601; doi:10.3389/fpls.2022.789701)
Supplement: Supplementary file 1 [file Data_Sheet_1.docx]

**Supplementary File 1.** List of alternative splicing forms of the two homoeologous *ASR* genes from chromosome group 4 of *Triticum durum* cv. Svevo (*TtASR-4A* and *TtASR-4B*), as annotated at INTEROMICS.

**>TRITD4Av1G160700.1**

ATGTCGGAGGAGAAGCACCACCACCTGTTCCACCACAAGGAGGGCGAGGACTTCCAGCCCGCCGCTGACGGCGGCGTCGACACGTACGGGTACTCGACCGAGACGGTGGTGACCGCCACCGGCAACGACGGCGAGTACGAGCGGATCACCAAGGAGGAGAAGCACCACAAGCACAAGGAGCACCTCGGCGAGATGGGCGCAGCCGCGGCCGGAGCCTTCGCCCTCTACGAGAAGCACGAGGCGAAGAAGGACCCGGAGCACGCGCACAAGCACAAGATCGAGGAGGAGGTGGCTGCCGCCGCAGCCGTCGGCGCCGGCGGCTTCGTCTTCCACGAGCACCACGAGAAGAAGCAGGACCACAAGGAGGCCAAGGAGGCCAGCGGCGAGAAGAAGCACCACCACTTCGGCTAG

**>TRITD4Av1G160700.2**

ATGTCGGAGGAGAAGCACCACCACCTGTTCCACCACAAGGAGGGCGAGGACTTCCAGCCCGCCGCTGACGGCGGCGTCGACACGTACGGGTACTCGACCAAGGAGGAGAAGCACCACAAGCACAAGGAGCACCTCGGCGAGATGGGCGCAGCCGCGGCCGGAGCCTTCGCCCTCTACGAGAAGCACGAGGCGAAGAAGGACCCGGAGCACGCGCACAAGCACAAGATCGAGGAGGAGGTGGCTGCCGCCGCAGCCGTCGGCGCCGGCGGCTTCGTCTTCCACGAGCACCACGAGAAGAAGCAGGACCACAAGGAGGCCAAGGAGGCCAGCGGCGAGAAGAAGCACCACCACTTCGGCTAG

**>TRITD4Av1G160700.3**

ATGTCGGAGGAGAAGCACCACCACCTGTTCCACCACAAGGAGGGCGAGGACTTCCAGCCCGCCACCGGCAACGACGGCGAGTACGAGCGGATCACCAAGGAGGAGAAGCACCACAAGCACAAGGAGCACCTCGGCGAGATGGGCGCAGCCGCGGCCGGAGCCTTCGCCCTCTACGAGAAGCACGAGGCGAAGAAGGACCCGGAGCACGCGCACAAGCACAAGATCGAGGAGGAGGTGGCTGCCGCCGCAGCCGTCGGCGCCGGCGGCTTCGTCTTCCACGAGCACCACGAGAAGAAGCAGGACCACAAGGAGGCCAAGGAGGCCAGCGGCGAGAAGAAGCACCACCACTTCGGCTAG

**>TRITD4Av1G160700.4**

ATGTCGGAGGAGAAGCACCACCACCTGTTCCACCACAAGGAGGGCGAGGACTTCCAGCCCGCCGCTGACGGCGGCGTCGACACGTACGGGTACTCGACCGAGACGGTGGTGACCGCCACCGGCAACGACGGCGAGTACGAGCGGATCACCAAGGAGGAGAAGCACCACAAGCACAAGGAGCACCTCGGCGAGATGGGCGCAGCCGCGGCCGGAGCCTTCGCCCTCTACGAGAAGCACGAGGCGAAGAAGGACCCGGAGCACGAGCACCACGAGAAGAAGCAGGACCACAAGGAGGCCAAGGAGGCCAGCGGCGAGAAGAAGCACCACCACTTCGGCTAG

**>TRITD4Av1G160700.5**

ATGTCGGAGGAGAAGCACCACCACCTGTTCCACCACAAGGAGGGCGAGGACTTCCAGCCCGCCGCTGACGGCGGCGTCGACACGTACGGGTACTCGACCGAGACGGTGGTGACCGCCACCGGCAACGACGGCGAGTACGAGCGGATCACCAAGTACGAGAAGCACGAGGCGAAGAAGGACCCGGAGCACGCGCACAAGCACAAGATCGAGGAGGAGGTGGCTGCCGCCGCAGCCGTCGGCGCCGGCGGCTTCGTCTTCCACGAGCACCACGAGAAGAAGCAGGACCACAAGGAGGCCAAGGAGGCCAGCGGCGAGAAGAAGCACCACCACTTCGGCTAG

**>TRITD4Av1G160700.6**

ATGTCGGAGGAGAAGCACCACCACCTGTTCCACCACAAGGAGGGCGAGGACTTCCAGCCCGCCGCTGACGGCGGCGTCGACACGTACGGGTACTCGACCGAGACGGTGGTGACCGCCACCGGCAACGACGGCGAGTACGAGCGGATCACCAAGGAGGAGAAGCACCACAAGCACAAGGAGCACCTCGGCGAGATGGGCGCAGCCGCGGCCGGAGCCTTCGCCCTCTACGAGAAGCACGAGGCGAAGAAGGACCCGGAGCACGCGCACAAGCACAAGATCGAGGAGGAGGTGGCTGCCGCCGCAGCCGTCGGCGCCGGCGGCTTCGTCTTCCACGAGCACCACGAGAAGAAGCAGGACCACAAGGAGGCCGTGCGTGTGCCTACGTTACGTGCGTTCCATAAGTGA

**>TRITD4Bv1G043860.1**

ATGGCGGAGGAGAAGCACCACCACCACCTGTTCCACCACAAGAAGGAGGGCGAGGACTTCCAGCCCGCCGCTGACGGCGGCGTCGACATGTACGGGTACTCGACCGAGACGGTGGTGACCGCCACCGGCAACGAGGGCGAGTACGAGCGGATCACCAAGGAGGAGAAGCACCACAAGCACAAGGAGCACCTCGGCGAGATGGGCGCCGCCGCCGCCGGAGCCTTCGCCCTCTACGAGAAGCACGAGGCGAAGAAGGACCCGGAGCACGCGCACAAGCACAAGATCGAGGAGGAGGTGGCCGCCGCCGCAGCCGTCGGCGCCGGTGGCTTCGTCTTCCACGAGCACCACGAGAAGAAGCAGGACCACAAGGAGGCCAAGGAGGCCAGCGGCGAGAAGAAGCACCACCACTTCGGCTAG

**>TRITD4Bv1G043860.2**

ATGGCGGAGGAGAAGCACCACCACCACCTGTTCCACCACAAGAAGGAGGGCGAGGACTTCCAGCCCGCCGCTGACGGCGGCGTCGACATGTACGGGTACTCGACCGAGACGGTGGTGACCGCCACCGGCAACGAGGGCGAGTACGAGCGGATCACCAAGGAGGAGAAGCACGAGGCGAAGAAGGACCCGGAGCACGCGCACAAGCACAAGATCGAGGAGGAGGTGGCCGCCGCCGCAGCCGTCGGCGCCGGTGGCTTCGTCTTCCACGAGCACCACGAGAAGAAGCAGGACCACAAGGAGGCCAAGGAGGCCAGCGGCGAGAAGAAGCACCACCACTTCGGCTAG

**>TRITD4Bv1G043860.3**

ATGGCGGAGGAGAAGCACCACCACCACCTGTTCCACCACAAGAAGGAGGGCGAGGACTTCCAGCCCGCCGCTGACGGCGGCGTCGACATGTACGGGTACTCGACCAAGGAGGAGAAGCACCACAAGCACAAGGAGCACCTCGGCGAGATGGGCGCCGCCGCCGCCGGAGCCTTCGCCCTCTACGAGAAGCACGAGGCGAAGAAGGACCCGGAGCACGCGCACAAGCACAAGATCGAGGAGGAGGTGGCCGCCGCCGCAGCCGTCGGCGCCGGTGGCTTCGTCTTCCACGAGCACCACGAGAAGAAGCAGGACCACAAGGAGGCCAAGGAGGCCAGCGGCGAGAAGAAGCACCACCACTTCGGCTAG

**>TRITD4Bv1G043860.4**

ATGGCGGAGGAGAAGCACCACCACCACCTGTTCCACCACAAGAAGGAGGGCGAGGACTTCCAGCCCGCCGCTGACGGCGGCGTCGACATGTACGGGTACTCGACCGAGACGGTGGTGACCGCCACCGGCAACGAGGGCGAGTACGAGCGGATCACCAAGGAGGAGAAGCACCACAAGCACAAGGAGCACCTCGGCGAGATGGGCGCCGCCGCCGCCGGAGCCTTCGCCCTCTACGAGAAGCACGAGGCGAAGAAGGACCCGGAGCACGAGCACCACGAGAAGAAGCAGGACCACAAGGAGGCCAAGGAGGCCAGCGGCGAGAAGAAGCACCACCACTTCGGCTAG

**>TRITD4Bv1G043860.5**

ATGGCGGAGGAGAAGCACCACCACCACCTGTTCCACCACAAGAAGGAGGGCGAGGACTTCCAGCCCGCCACCGGCAACGAGGGCGAGTACGAGCGGATCACCAAGGAGGAGAAGCACCACAAGCACAAGGAGCACCTCGGCGAGATGGGCGCCGCCGCCGCCGGAGCCTTCGCCCTCTACGAGAAGCACGAGGCGAAGAAGGACCCGGAGCACGCGCACAAGCACAAGATCGAGGAGGAGGTGGCCGCCGCCGCAGCCGTCGGCGCCGGTGGCTTCGTCTTCCACGAGCACCACGAGAAGAAGCAGGACCACAAGGAGGCCAAGGAGGCCAGCGGCGAGAAGAAGCACCACCACTTCGGCTAG
