# Supplementary material for: Abscisic Acid-Stress-Ripening Genes Involved in Plant Response to High Salinity and Water Deficit in Durum and Common Wheat
Source: Front Plant Sci. 2022 Feb 16;13:789701. doi: 10.3389/fpls.2022.789701 (PMC8905601; doi:10.3389/fpls.2022.789701)
Supplement: Supplementary file 2 [file Data_Sheet_2.docx]

**Supplementary File 2.** List of homoeologous *ASR* genes from chromosome group 4 of *Triticum aestivum* cv. Chinese Spring (*TaASR-4A*, *TaASR-4B*, *TaASR-4D*), along with the corresponding codified proteins, as annotated at EnsemblPlants database. Letters in bold are exons.

**> TraesCS4A02G208400 (*TaASR-4A*)**

**ATGTCGGAGGAGAAGCACCACCACCTGTTCCACCACAAGGAGGGCGAGGACTTCCAGCCCGCCGCTGACGGCGGCGTCGACACGTACGGGTACTCGACCGAGACGGTGGTGACCGCCACCGGCAACGACGGCGAGTACGAGCGGATCACCAAGGAGGAGAAGCACCACAAGCACAAGGAGCACCTCGGCGAGATGGGCGCAGCCGCGGCCGGAGCCTTCGCCCTC**GTACGCCCTCTCTCATCGTAACTAGGAGTAGTAATTACCATACGAATATATAGCTCTTGTCGGGCTTGGCCTAATGGATTGCGTGTCTACGTGCAG**TACGAGAAGCACGAGGCGAAGAAGGACCCGGAGCACGCGCACAAGCACAAGATCGAGGAGGAGGTGGCTGCCGCCGCAGCCGTCGGCGCCGGCGGCTTCGTCTTCCACGAGCACCACGAGAAGAAGCAGGACCACAAGGAG**GCCAAGGAGGCCAGCGGCGAGAAGAAGCACCACCACTTCGGCTAGGTCGCCGTCGACGTGCGGTGGCCGGCCTCGCCGCCG**GCCGTGCGTGTGCCTACGTTACGTGCGTTCCATAAGTGA**

**>A0A3B6HYQ0**

MSEEKHHHLFHHKEGEDFQPAADGGVDTYGYSTETVVTATGNDGEYERITKEEKHHKHKEHLGEMGAAAAGAFALYEKHEAKKDPEHAHKHKIEEEVAAAAAVGAGGFVFHEHHEKKQDHKEAVRVPTLRAFHK

**> TraesCS4B02G112000 (*TaASR-4B*)**

**ATGGCGGAGGAGAAGCACCACCACCACCTGTTCCACCACAAGAAGGAGGGCGAGGACTTCCAGCCCGCCGCTGACGGCGGCGTCGACATGTACGGGTACTCGACCGAGACGGTGGTGACCGCCACCGGCAACGAGGGCGAGTACGAGCGGATCACCAAGGAGGAGAAGCACCACAAGCACAAGGAGCACCTCGGCGAGATGGGCGCCGCCGCCGCCGGAGCCTTCGCCCTC**GTACGCTCTCTCTCATCATAACTAGTGGAGTCGTAATTACCATACGCATATATAGCTCTTGTCGGGCTTGGCTAATGGACTGCGTGTCTACGTGCAG**TACGAGAAGCACGAGGCGAAGAAGGACCCGGAGCACGCGCACAAGCACAAGATCGAGGAGGAGGTGGCCGCCGCCGCAGCCGTCGGCGCCGGCGGCTTCGTCTTCCACGAGCACCACGAGAAGAAGCAGGACCACAAGGAGGCCAAGGAGGCCAGCGGCGAGAAGAAGCACCACCACTTCGGCTAG**

**>A0A446R3P1**

MSEEKHHHLFHHKEGEDFQPAADGGVDTYGYSTETVVTATGNDGEYERITKEEKHHKHKEHLGEMGAAAAGAFALYEKHEAKKDPEHAHKHKIEEEVAAAAAVGAGGFVFHEHHEKKQDHKEAKEASGEKKHHHFG

**> TraesCS4D02G109500 (*TaASR-4D*)**

**ATGGCGGAGGAGAAGCACCACCACCACCTGTTCCACCACAAGGAGGGCGAGGACTTCCAGCCCGCCGCTGACGGCGGCGTCGACACGTACGGGTACTCGACCGAGACGGTGGTGACCGCCACCGGCAACGAGGGGGAGTACGAGCGGATCACCAAGGAGGAGAAGCACCACAAGCACAAGGAGCACCTCGGCGAGATGGGCGCCGCCGCCGCCGGAGCCTTCGCCCTC**GTACGCTCTCTCTCACCATAACTAATTACCGTACGCACATATAGCTCTTGTTGGGCTTGGCTAATAATGGACTGCGTGTCTACGTGCAG**TACGAGAAGCACGAGGCGAAGAAGGACCCGGAGCACGCGCACAAGCACAAGATCGAGGAGGAGGTGGCCGCCGCTGCAGCCGTCGGCGCCGGCGGCTTCGTCTTCCACGAGCACCACGAGAAGAAGCAGGACCACAAGGAGGCCAAGGAGGCCAGCGGCGAGAAGAAGCACCACCACTTCGGCTAG**

**>J7EJB7**

MAEEKHHHHLFHHKEGEDFQPAADGGVDTYGYSTETVVTATGNEGEYERITKEEKHHKHKEHLGEMGAAAAGAFALYEKHEAKKDPEHAHKHKIEEEVAAAAAVGAGGFVFHEHHEKKQDHKEAKEASGEKKHHHFG
