# Supplementary material for: Abscisic Acid-Stress-Ripening Genes Involved in Plant Response to High Salinity and Water Deficit in Durum and Common Wheat
Source: Front Plant Sci. 2022 Feb 16;13:789701. doi: 10.3389/fpls.2022.789701 (PMC8905601; doi:10.3389/fpls.2022.789701)
Supplement: Supplementary file 4 [file Table_2.DOCX]

**Supplementary Figure 2. (A)** Comparison between *TtASR1* gene isolated in the Tunisian durum wheat landrace Mahmoudi (Hamdi et al., 2020) and *TtASR-4B* gene of durum cv. Svevo annotated at INTEROMICS. Intron sequence is highlighted in grey; SNPs are highlighted in yellow. Underlined in red are reported the start and stop codons; underlined in dark blue are reported the splice junctions. **(B)** Alignment between predicted ASR protein of Mahmoudi (ASC55656.1) and ASR protein encoded by Svevo gene transcript TRITD4Bv1G043860.1. Amino acid substitutions are highlighted in yellow (same physico-chemical category) or red (different physico-chemical category); underlined is the conserved ABA_WDS domain. Alignments were performed by MUSCLE.


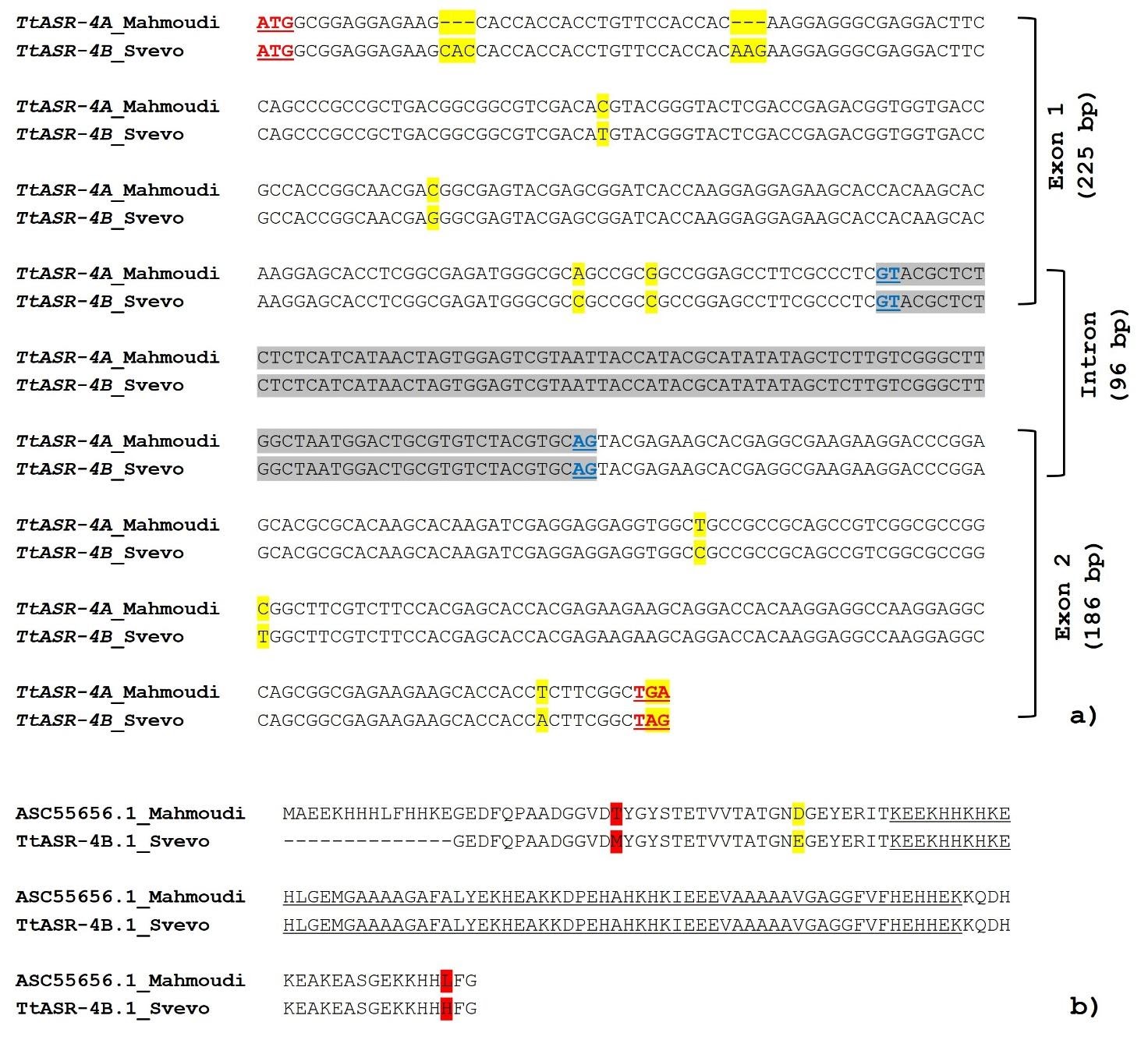


**(A)**

**(B)**
