# Supplementary material for: Abscisic Acid-Stress-Ripening Genes Involved in Plant Response to High Salinity and Water Deficit in Durum and Common Wheat
Source: Front Plant Sci. 2022 Feb 16;13:789701. doi: 10.3389/fpls.2022.789701 (PMC8905601; doi:10.3389/fpls.2022.789701)

**Supplementary Figure 3.** Multiple alignment of CDS from *TtASR-4A* gene of Tunisian durum wheat landrace Mahmoudi and the 6 splicing variants of *TtASR-4A* gene of *T. durum* cv. Svevo (TRITD4Av1G160700). The match with the highest similarity (99%) is reported in red. SNPs are highlighted in yellow.


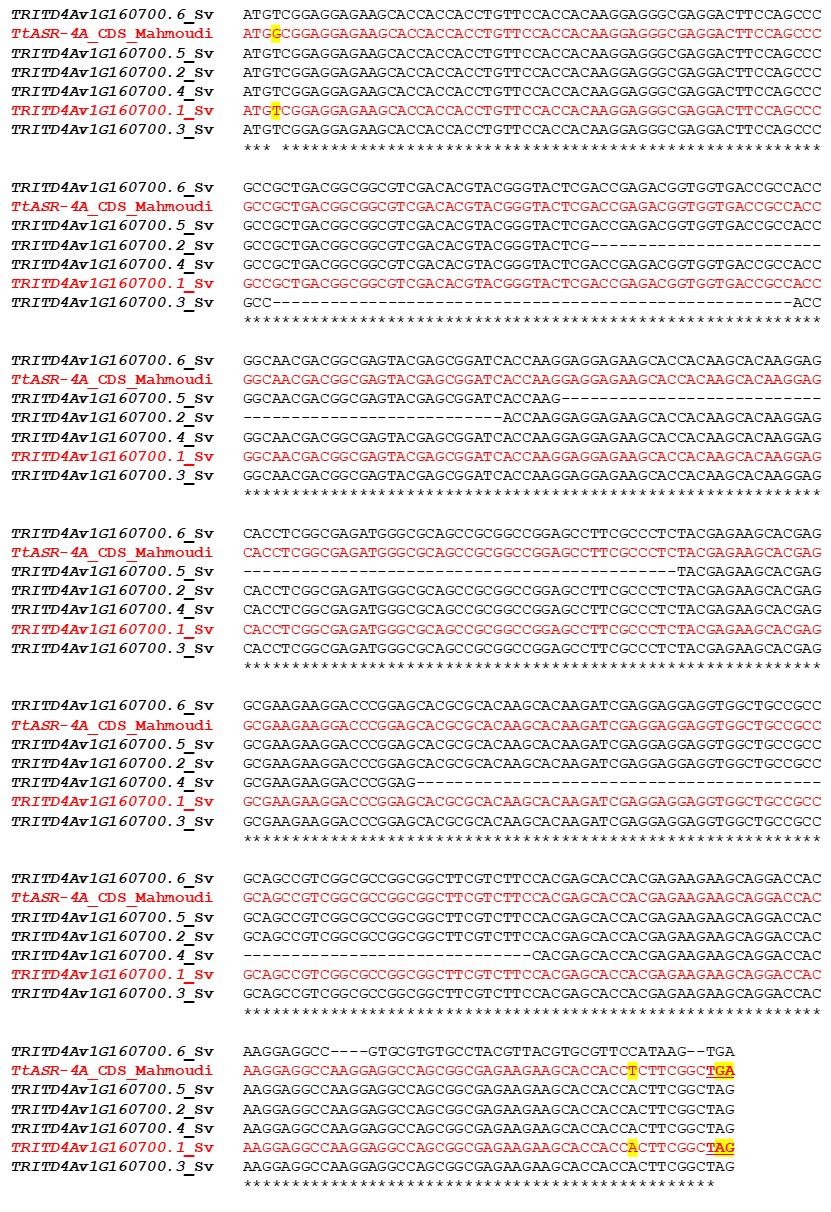

Supplement: Supplementary file 5 [file Table_3.DOCX]
