# Supplementary material for: Abscisic Acid-Stress-Ripening Genes Involved in Plant Response to High Salinity and Water Deficit in Durum and Common Wheat
Source: Front Plant Sci. 2022 Feb 16;13:789701. doi: 10.3389/fpls.2022.789701 (PMC8905601; doi:10.3389/fpls.2022.789701)

**Supplementary Figure 4.** Preliminary qRT-PCR amplification assay with fluorescent SYBRGreen dye of *TaASR-4B/4D* genes in common wheat cv. Ta002^S^, using P2 primer pair. **(A)** Testing of primer concentration in a gradient of 300 nM (blue), 500 nM (red) and 900 nM (green). Curves represent relative fluorescence RFU (y-axis) respect to cycle number (x-axis). Arrow indicates optimal primer concentration giving the best amplification curve, lowest Ct and highest endpoint RFU. **(B)** Optimization of annealing temperature: primer specificity is showed by melting curves of PCR products.


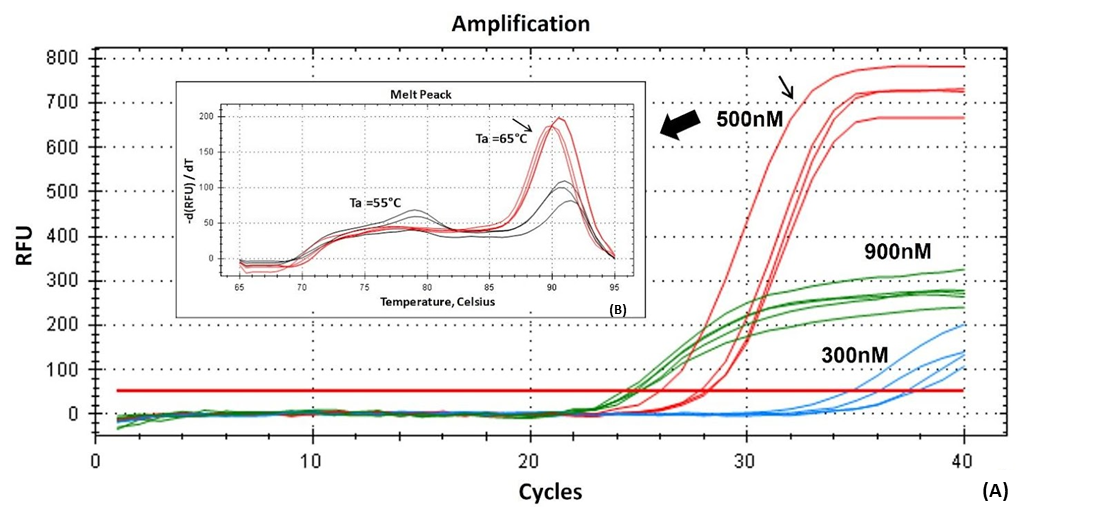

Supplement: Supplementary file 6 [file Table_4.DOCX]
